# Annotations

**Published:** 1888-01-07

**Authors:** 


### ANNOTATIONS.

**"As they Face the Billows."**

MOST people are familiar with "Caller Herrin," the pathetic song of which the Baroness Nairne was the well-known authoress. Little as we who "sit at home at ease" think of the fish which gives variety to our tables; and "vulgar," as the fishwife complains, we specially regard the herring as being, yet "wives and mithers maist despairing, call them 'lives of men.'" And "lives of men" the deep sea-fish also may often be called with simple truth. "Twelve thousand men, without one doctor in their midst." Such is the plain statement of fact with regard to the smacksmen of the North Sea. For eight weeks at a stretch those twelve thousand are cut off entirely from all home ties and comforts, and exposed to constant and grave peril to life and limb. "They labour night and day amid furious storms and bitter cold, and the cases of illness and accident—fractures, contusions, etc., are so numerous as to simply appal an average landsman." For some years there was absolutely no succour of any kind for the poor fellows, and patients suffering from severe compound fractures were often perforce detained for a week, and in one instance thirteen days, before surgical treatment could be obtained. It is no wonder that the sufferings of those hardy heroes were terrible, whilst the loss of life was enormous. The Mission to Deep-sea Fishermen has equipped eight vessels to follow the trawlers, each fitted with a medicine compartment and a surgical chest. Last year nearly four thousand out of a total of twelve thousand individuals, were patients. One in every four of all those able-bodied men struck down with illness or accident. Probably no calling in the world is more arduous and dangerous than that of deep-sea fishermen. What possible boon could be so welcome to them as that which it is proposed to send with their daring fleet? The project is to build and equip a floating hospital, to be placed under the charge of a skilful and courageous surgeon. It will surprise no one that her Majesty the Queen has graciously signified her deep interest in the scheme. Not only so, but she has consented to become the patron of the mission, and has contributed handsomely to the fund being raised for the building of the ship. The vessel when finished will bear the honoured name of *Queen Victoria*. Need anything more be given than this bare recital of facts to make every brave-hearted man and woman, and even child, in Britain resolve to have a share, however small, in the building and furnishing of this hospital ship? All who propose to have a plank in it should make known their wishes to the Secretary, at 181, Queen Victoria Street, E.C.

**Suffering Children.**

WE assumed in last week's issue that everyone means well to children; that though people may err in judgment their affection is not at fault. But the report of the Society for Preventing Cruelty to Children shows that there are at least a few to whom the helplessness of childhood does not appeal, and who seem incapable of those natural affections which we term instinctive because we share them with the animals whom we consider destitute of reason. It seems an insult to the sacred names of father and mother to apply them to the parents spoken of in this report. It is impossible to detail in these pages all the cruelties reported, but a few may be mentioned. Here is a case of a child being left to sit on the bare oil-cloth of a passage, in a thorough draught, shivering and ill, dressed in nothing but a night-gown. Within a room opening from this very passage, its mother, who had left it there, sat before a good fire eating a comfortable breakfast. The reason of her cruelty may be stated in a few words: The child's life was insured for £7; therefore its death was desired. In another case, a father sent his little daughter upstairs to prepare for a whipping; meanwhile he, being too tired at the moment to execute his task with sufficient severity, lay down on the sofa to recruit. While he was resting, the child, mad with fright, threw herself out of the window and was killed. By some peculiarity of the law it was impossible to punish this self-indulgent torturer. Children who are naturally weak or afflicted suffer even more than others from those who have control of them, as witness the instances of a deaf and dumb boy being beaten because it was "so difficult to make him understand," and a red-hot poker drawn before the eyes of a blind girl and her hands being touched with it—"for fun!" The society for preventing these and worse cruelties, and rescuing the victims from their inhuman guardians, has been in existence only three years, during which time it has interfered on behalf of nearly 900 children; but, like other charitable bodies, it requires pecuniary aid to pursue its work, both in prosecuting the brutal parents and guardians, and in finding other homes, either in this country or abroad, for the children—at least, those of them who survive their sufferings. The Baroness Burdett-Coutts, who writes the preface to the report, pleads the cause of the society. We need say nothing of its value; it is sufficiently obvious. The office of the society is at 7, Harpur Street, W.C., and its Secretary is The Rev. Benjamin Waugh, who will gladly receive subscriptions.

**Epilepsy and Responsibility.**

BENJAMIN HEWLETT, a police-constable, forty-one years of age, was charged at the Westminster Police-court last week with feloniously cutting and wounding his son Ernest, aged nine, with a chopper. According to the evidence offered, Hewlett went to the Gerald Road Police Station in a very excited condition, and, looking wildly about, said, "I am going mad; I have killed my boy." Inspector Curtis, on visiting his home, found the boy bleeding from two very severe wounds in the head. On a table in an adjoining room was a chopper still wet with blood. There was also a large quantity of blood on the floor of the room where the boy was found, and the pages of a school-book which he had been reading were likewise splashed with blood. The prisoner, when he was first seen at the police-station, was perfectly sober. Dr. Neville found two deep wounds, each about three inches long, on the boy's head, and in both cases the blow had been so heavy as not only to cut through the scalp, but to fracture the skull. At the time when the charge was made the child was still alive, though very feeble. It was proved that the prisoner had suffered from epileptic fits for many years. We do not publish afresh the details of this terrible attempt on the part of a parent to murder his own child for the purpose of harrowing our readers' feelings, but to point out that such a dreadful tragedy should not have been possible. Most medical men know of families in which there are epileptics who are permitted to mix freely with the other members of the household, and who periodically give way to fits of violent excitement, in which they might murder not one but several people before anything could be done to prevent them. Such persons are both responsible and irresponsible—responsible when calm, and irresponsible when under the influence of excitement. When calm they are not dangerous, and seldom or never do anything which calls for severe punishment. But if an epileptic, in a fit of frenzy, should kill another person, what is the degree of responsibility? There is no responsibility at all on the part of the epileptic, but only on the part of those who permitted him to be in unrestrained liberty. The moral of this, as of many similar stories, is that pronounced epileptics ought not to be permitted to be at large. They should, if kept at home, be placed under the charge of some responsible person; and where such a course is for any reason impracticable, they should be taken charge of in public institutions specially set apart for that purpose. The want of such institutions is exceedingly and increasingly urgent at the present time; and if private benevolence does not supply the want, the Local Government Board should feel itself imperatively bound to do so.

